# Three new species of cockroach genus *Symploce* Hebard, 1916 (Blattodea, Ectobiidae, Blattellinae) with redescriptions of two known species based on types from Mainland China

**DOI:** 10.3897/zookeys.337.5770

**Published:** 2013-09-30

**Authors:** Zongqing Wang, Yanli Che

**Affiliations:** 1 Institute of Entomology, College of Plant Protection, Southwest University, Beibei, Chongqing 400716,China

**Keywords:** Insecta, Dictyoptera, Ectobiidae, *Episymploce*, new species, cockroaches

## Abstract

Three new species of *Symploce* Hebard from China are described: *Symploce sphaerica* **sp. n.**, *Symploce paramarginata* **sp. n.** and *Symploce evidens* **sp. n.** Two known species are redescribed and illustrated based on types. A key is given to identify all species of *Symploce* from mainland China.

## Introduction

The genus *Symploce* was erected by Hebard in 1916, and it is the most primitive genus of the family Ectobiidae, for related to the earliest fossil species *Pinblattella citimica* according to Vršanský (1997). Princis (1969) lists 109 species of this genus in *Orthopterorum Catalogus*, among which 23 species were known from China. Asahina (1974) added 2 species named *Symploce miyakoensis* and *Symploce okinoerabuensis* from Japan, and in 1979, he described 2 species *Symploce yayeyamana*, *Symploce gigas* and 2 subspecies *Symploce gigas okinawana*, *Symploce striata wulai*; transferred *Parasymploce accuminata* (Shiraki, 1931) and *Ischnoptera furcata* Shiraki, 1931 to *Symploce* as well. Kumar (1975) reported 3 species, *Symploce medleri*, *Symploce marshallae*, *Symploce distincta*, from Africa. Feng and Woo (1988, 1993, 1999) reported *Symploce wulingensis*, *Symploce mamillatus*, *Symploce guizhouensis* species from Southern China; Woo and Feng published two species *Symploce bispota* and *Symploce quadripinis*, respectively in 1988 and 1992. In 2002, Feng described a species of *Symploce*, named *jianfengensis* from China. Izquierdo and Medina (1992) reported one species from Gran Canaria, Spain. Roth (1984a) redefined the generic characters of *Symploce* and redescribed 9 species from West Indies and a key was given for the males. In the same year, Roth (1984b, 1984c) described 13 species from New Guinea, 5 species and 3 known species from Borneo, Flores, India and Philippines, and defined groups for New Guinea species; in addition, keys were given based on examined males. In 1985a he described 6 species, redescribed 2 known species from Borneo, Sumatra and West Malaysia, a key was given to the males; In 1985b he described four new species and redescribed five species, and brief descriptions of three known species were given from Bey-Bienko’s original description; In 1986a, b he described 8 species and redescribed 14 known species from African and divided them into four groups, a key to males is given. In 1987a Roth redescribed 6 species and 4 subspecies from Taiwan and Japanese Islands. A key to males and a worldwide distribution checklist of all species of the genus were given. Roth (1991) transferred *Symploce triramosa* (Saussure, 1869) and *Symploce tchadiana* Roth, 1987 to *Carbrunneria* Princis and *Supella* Rehn respectively. Roth (1997) synonymized 2 species, *Symploce bicolorata* Roth, 1985 with *Haplosymploce montis* (Shelford, 1906) and *Symploce ferruginea* Roth, 1985 with *Haplosymploce nigra* (Hanitsch, 1928) and transferred 12 species to *Episymploce*. And in 1999, Roth described a species, *Symploce stupida*. During the period of revising the genus *Symploce*, Roth reassigned many species; among them, most Chinese species listed in Princis’ Catalogue (except *Symploce striata*) were related and transferred to *Episymploce* Bey-Bienko. Subsequently Roth (2003) transferred 2 species, namely, *Symploce guizhouensis* and *Symploce mamillatus*, described by Feng and Woo (1988) to *Episymploce* based on the drawings of the supra-anal and subgenital plates. Up to now, only 65 species were included in this genus worldwide (Beccaloni 2007), of which 8species are from China (including Taiwan) but just 5 species are distributed in mainland China, one of which, *Symploce dimorpha* has been transferred into *Episymploce* by Roth (1987b).

In the present paper, after checking the specimens deposited in the Insect Collection of Southwest University (SWU), 3 species new to science are described and illustrated and 2 known species based on types are redescribed.

## Materials and methods

Terminology used in this paper is mainly according to Roth (2003). Measurements are based on specimens examined. The genital segments of the examined specimens were macerated in 10% NaOH and observed in glycerin jelly using a Motic K400 stereomicroscope. All the drawings were made with the aid of a Motic K400 stereomicroscope. Photographs of the specimens were made using a Canon 50D plus a Canon EF 100mm f/2.8L IS USM Macro lens with the aid of the Helicon Focus software. The type specimens are deposited in the Insect Collection of Southwest University, Beibei, China.

## Taxonomy

### Symploce

Hebard, 1916

http://species-id.net/wiki/Symploce

Symploce Hebard, 1916: 355; Roth 1984a: 26.

Type species.

*Blatta capitata* Saussure, 1862.

#### Diagnosis.

Tegmina and wings fully developed or more or less reduced. Radius vein of hind wing unbranched or branched near the middle; cubitus vein straight to distinctly curved with 1-6 complete and 0-6 incomplete branches; apical triangle absent or present, small or large. Specialization on the male abdominal tergum varies considerably in position and shape or is completely unmodified. Supra-anal plate symmetrical, rarely asymmetrical. Subgenital plate weakly to strongly asymmetrical, rarely symmetrical, with various highly specialized styli. Anteroventral margin of front femur usually Type A_3_, rarely Type B_3_ or between Type A and B. Tarsal claws symmetrical, unspecialized.

#### Note.

It is proposed that *Symploce quadrispinis* Feng & Woo, *Symploce stellatus* Feng & Woo, 1999 and *Symploce wulingensis* Feng & Woo should be transferred to *Episymploce*. Plus one known species, *Symploce jianfengensis* Feng, 2002, which was not recorded in the catalogue by Beccaloni (2007), also should be transferred to *Episymploce* (Wang at al. in press).

#### Distribution.

Oriental, Australian, African and Palaearctic Regions.

#### Key to species of *Symploce* from Mainland China (Males)

**Table:** 

1	The first abdominal tergum unmodified	2
–	The first abdominal tergum modified	3
2	Pronotum with two black longitudinal, irregular maculae centrally, supra-anal plate linguiform	*Symploce bispot*
–	Pronotum with two small V- shaped brown spots centrally, supra-anal plate trapeziform	*Symploce torchaceus*
3	Seventh abdominal tergum modified with a pair of large depressions where some setae are situated	*Symploce sphaerica* sp. n.
–	Seventh abdominal tergum unmodified	4
4	Pronotum pitch black, lateral board brown	*Symploce paramarginata* sp. n.
–	Pronotum yellowish brown with shallow U-shaped dark brown macula near base	*Symploce evidens* sp. n.

### 1. Symploce torchaceus

Feng & Woo, 1999

http://species-id.net/wiki/Symploce_torchaceus

Figs 1–2 , 11–19

Symploce torchaceus Feng & Woo, 1999: 51.

#### Description.

**Figures 1–10.**
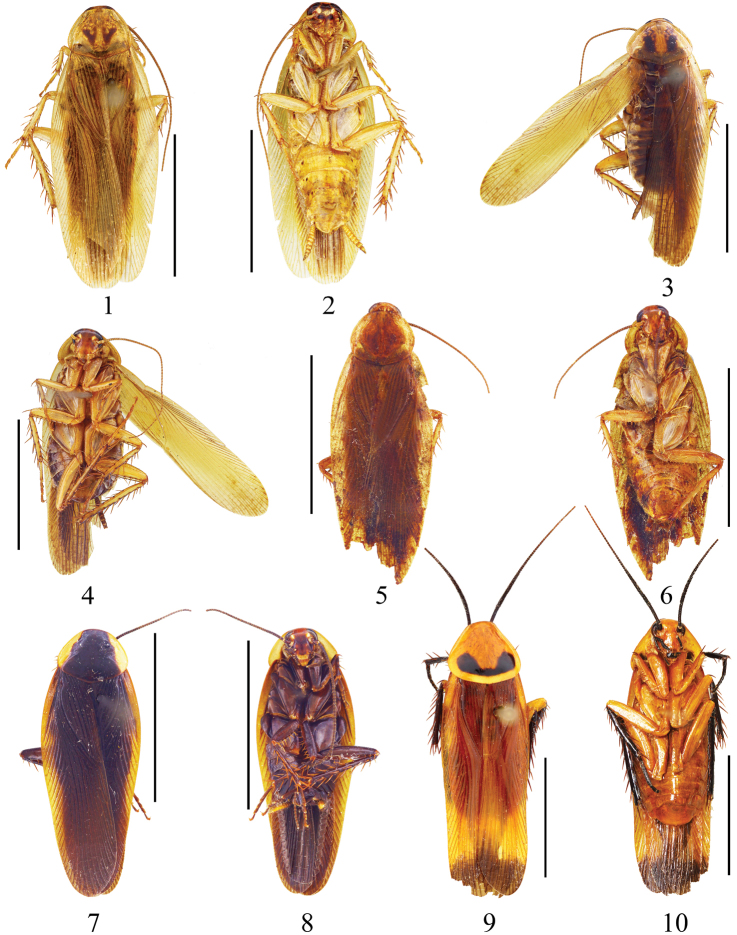
**1–2**. *Symploce torchaceus* Feng & Woo, male: **1** holotype, dorsal view **2** same, ventral view **3–4** *Symploce bispot* Feng and Woo, male: **3** holotype, dorsal view **4** same, ventral view **5–6** *Symploce sphaerica* sp. n., male: **5** holotype, dorsal view **6** same, ventral view **7–8** *Symploce paramarginata* sp. n., male: **7** holotype, dorsal view **8** same, ventral view **9–10** *Symploce evidens* sp. n., male: **9** holotype, dorsal view **10** same, ventral view. Scale bars = 1.0 cm.

**Figures 11–19.**
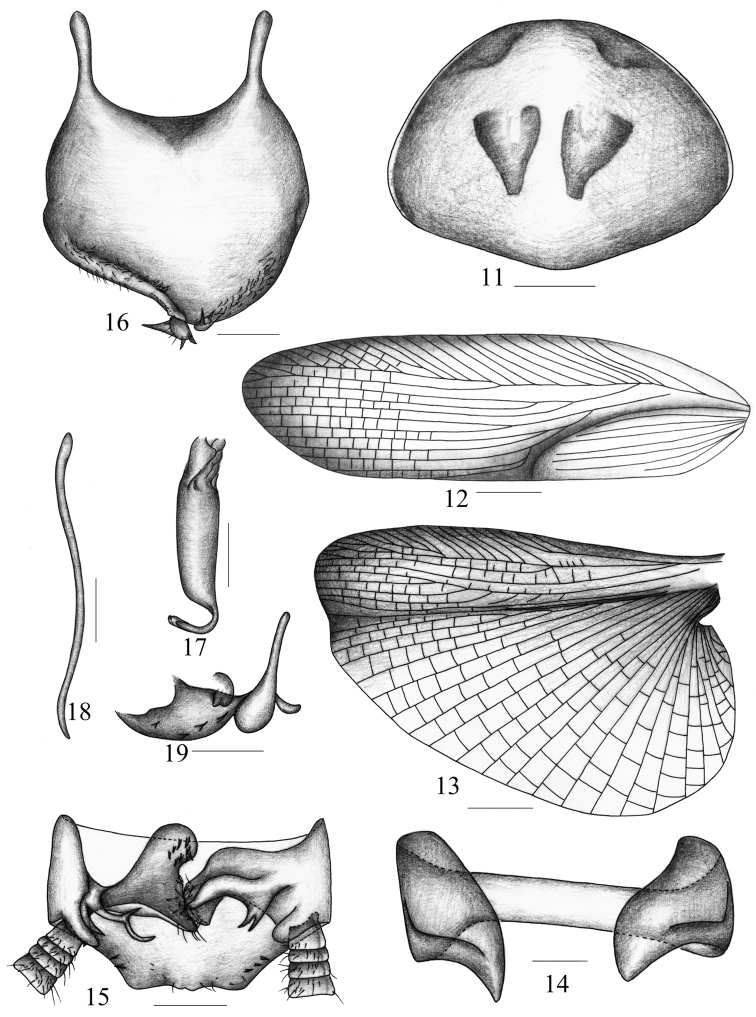
*Symploce torchaceus* Feng and Woo **11** pronotum **12** tegmen **13** hind wing (vannal region damaged) **14** abdominal tergum 9 and lateral plates, ventral view **15** supra-anal plate and paraprocts, ventral view **16** subgenital plate, dorsal view **17** hook-like phallomere **18** median phallomere **19** right phallomere. Scale bars = 1.0 mm (Fig. 11), 2.0 mm (Figs 12–13), 0.5 mm (Figs 14–19).

Length, male,pronotum: length × width: 3.2 × 3.8mm, tegmen: 15mm, overall length: 18mm. Body brown (Fig. 1). Head brown with a dark brown band on disc, which is wide and short. Face brown with a dark brown band. Maxillary palpomus brown and apex dark brown (Fig. 2). Pronotum pale brown with a pair of V-shaped rufous maculae in centre (Figs 1, 11). Tergum (except T_1_) with a dark brown spot on each side, and a dark brown stripe on disk.

Vertex with interocular width slightly less than ocellus width, distinctly narrower than distance between antennal sockets. Fourth and fifth maxillary palpomus of same length, both distinctly shorter than third (Fig. 2). Pronotum elliptical and width longer than length, with anterior margin nearly truncate and hind margin slightly produced in the middle (Fig. 11). Tegmen and hind wing well developed, entirely covering abdomen (Fig. 1). Tegmen narrow and long; radius vein with apical posterior branch, which terminates at the apical margin, and with 2 small branches, one of them branched; median vein with 2 branches (Fig. 12). Radius vein of hind wing branched beyond the middle and the branches bifurcated again near apex, median vein slightly curved and simple; cubitus slightly curved with 3 complete and 3-4 incomplete branches, triangular apical area reduced and small (Fig. 13). Anteroventral margin of front femur type A_3_, pulvilli present on 4 proximal tarsomeres, tarsal claws symmetrical and unspecialized, and arolia present. The 1st abdominal tergum (T_1_) unmodified, T_7_ specialized with some setae on disc; lateral plates of T_9_ similar with hind margin rounded and unspecialized (Fig. 14).

**Male genitalia.** Supra-anal plate (Fig. 15) in ventral view symmetrical and nearly trapeziform, hind margin nearly straight, left side with 3 small spines and right side with 2 small spines. Right and left paraprocts (Fig. 15) obviously asymmetrical, left one dendritic and apices tapering, right one with apex scattered with many fine spines and 1 branch near base, which resembles an antler. Subgenital plate (Fig. 16) weakly asymmetrical and hind margin slightly produced in the middle, left side concave at apical half and right side curved; two styli dissimilar and lying at apex, left style large which is similar to one torch directed laterad, and with 3 small teeth at outer margin near base, right style smaller and with 3 acute spines at apex. Hook of left phallomere with sclerotized portion very small, on left side, slender and with V-shaped incision (Fig. 17). Median phallomere (Fig. 18) long and lanciform with apex tapering, right phallomere (Fig. 19) skilletlike with a twist of sclerite.

#### Materials examined.

One male (holotype), China: Fujian Prov., Mt. Wuyishan, 10 July 1982, coll. Feng Xia; one male (paratype), China: Hainan Prov., Mt. Jianfengling, Tianchi, 21 March 1983, coll. Shaoying Liang; one male, China: Hainan Prov., Mt. Jianfeng, 12 March 1982, coll. Maobin Gu; four males, China: Hainan Prov., Mt. Jianfengling, 25 March 1985, coll. Zhiqing Chen.

#### Distribution.

China (Fujian, Hainan).

### 2. Symploce bispot

Feng & Woo, 1988

http://species-id.net/wiki/Symploce_bispot

Figs 3–4 , 20–27

Symploce bispot Feng & Woo, 1988: 31.

#### Description.

**Figures 20–27.**
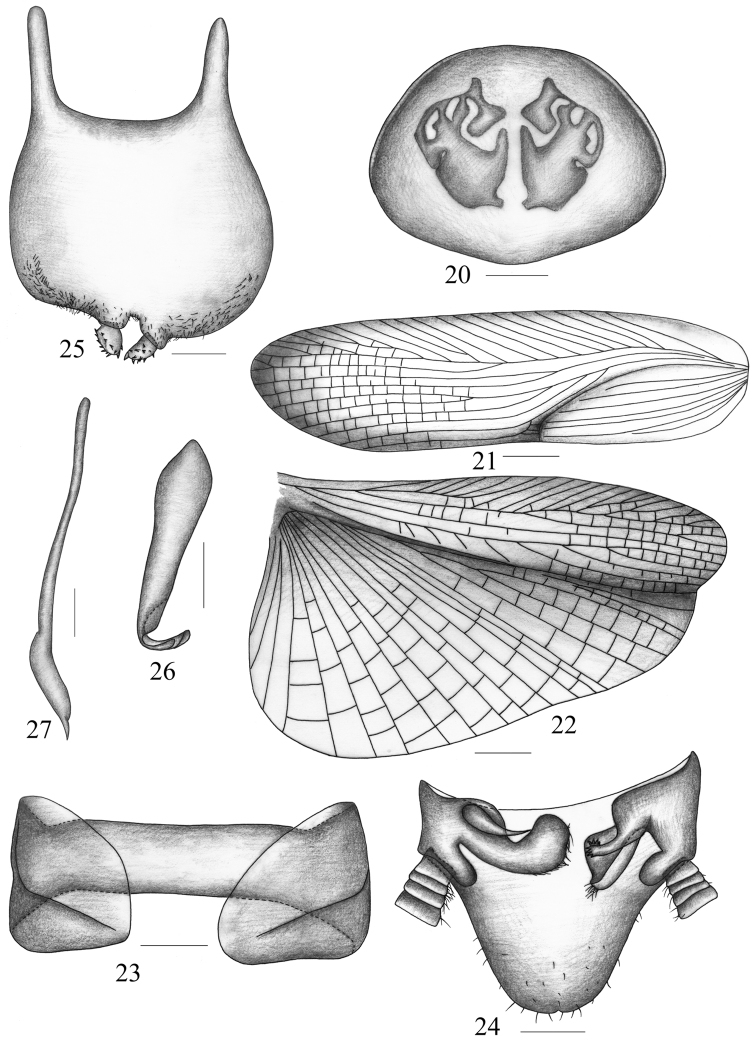
*Symploce bispot* Feng & Woo (right phallomere missing) **20** pronotum **21** tegmen **22** hind wing **23** abdominal tergum 9 and lateral plates, ventral view **24** supra-anal plate and paraprocts, ventral view **25** subgenital plate, dorsal view **26** hook-like phallomere **27** median phallomere. Scale bars = 1.0 mm (Fig. 20), 2.0 mm (Figs 21–22), 0.5 mm (Figs 23–27).

Length, male,pronotum: length × width: 4.0 × 5.0mm, tegmen: 19.0mm, overall length: 19.5–20.0mm. Body brown (Fig. 3). Head brown with area between and beyond ocellus reddish brown. Occiput region pale yellow. Antenna brown with base yellowish brown and apex dark brown. Maxillary palpomus yellowish brown and apical segment blackish brown (Fig. 4). Pronotum brown with dark brown maculae on disc (Figs 3, 20). Tergum reddish brown and both sides pale brown.

Vertex with interocular width slightly narrower than ocellus width, distinctly less than distance between antennal sockets. Fourth and fifth maxillary palpomus about same length, both distinctly shorter than the third (Fig. 4). Pronotum more or less elliptical, with anterior margin nearly truncate and hind margin obviously produced in the middle; pronotum with irregular maculae as Fig. 20 on disc. Tegmen and hind wing well developed, entirely covering abdomen. Tegmen narrow and long; radius vein with apical posterior branch, which bifurcated at apical part; median vein branched near the middle (Fig. 21). Radius vein of hind wing branched around the middle and apex of branch bifurcated, median vein simple; cubitus vein with 3 complete and 4 incomplete branches, triangular apical area small (Fig. 22). Anteroventral margin of front femur Type A_3_, pulvilli present on 4 proximal tarsomeres, tarsal claws symmetrical and unspecialized, and arolia present. T_1_, T_7_ unmodified, 1 semitransparent spot present on disc of T_3_, T_4_, T_5_, T_6_. Lateral plate of T_9_ about similar and with hind margin unproduced and unspecialized (Fig. 23).

**Male genitalia.** Supra-anal plate in ventral view symmetrical and about linguiform (Fig. 22). Right and left paraprocts (Fig. 24) evidently asymmetrical, left one dendritic and apices tapering, right one with apex scattered with lots of fine spines and 1 branch near base, which resembles an antler. Subgenital plate (Fig. 25) slightly asymmetrical, hind margin slightly concave in the middle and produced, where with two styles lying at both sides; style nearly elliptical and with small spines at outer margin. Hook of left phallomere with sclerotized portion very small, on left side, slender and with V-shaped incision (Fig. 26). Median phallomere long and lanciform, with apex tapering and rarely curved near apex (Fig. 27).

Female similar to male. Supra-anal plate triangular, subgenital plate broad and round.

#### Materials examined.

One male (holotype), China: Xizang Prov., 23 November 1983, coll. Yinheng Han; one female (paratype), same data as holotype; one male, China: Xizang Prov., Beibeng, 13 December 1977, coll. Jianshe Wu.

#### Distribution.

China (Xizang).

### 3. Symploce sphaerica sp. n.

http://zoobank.org/54B15C64-8C73-4A8B-A6AA-185AB2107546

http://species-id.net/wiki/Symploce_sphaerica

Figs 5–6 , 28–37

#### Description.

**Figures 28–37.**
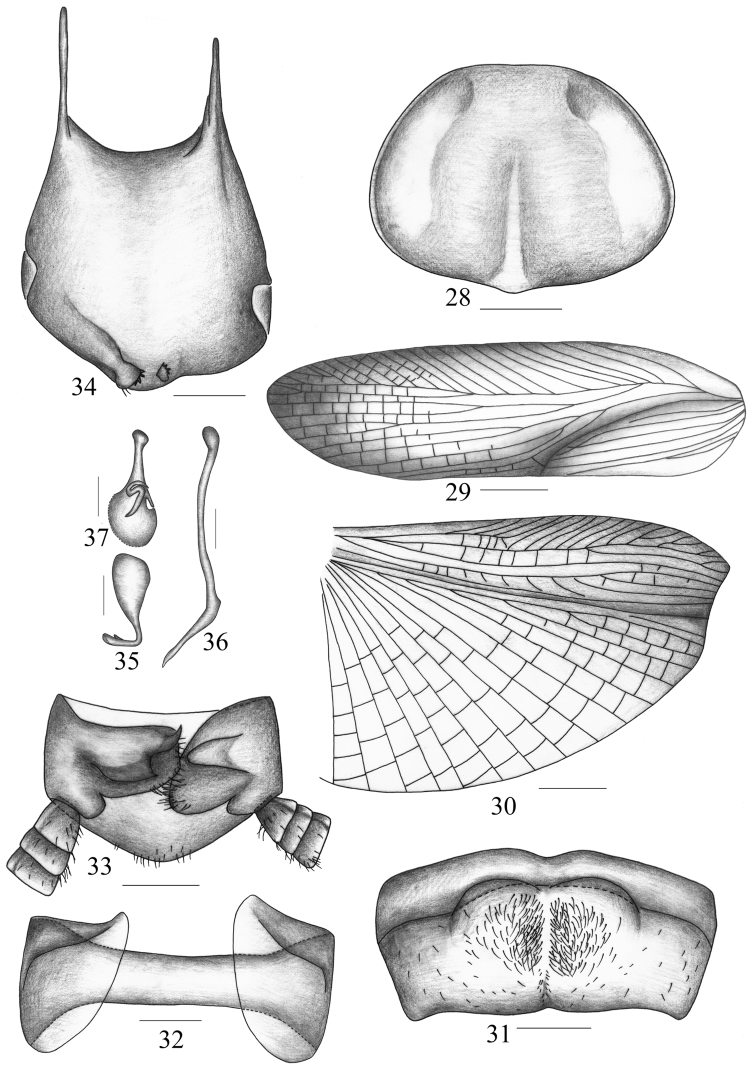
*Symploce sphaerica* sp. n. **28** pronotum **29** tegmen **30** hind wing **31** abdominal tergum 7, dorsal view **32** abdominal tergum 9 and lateral plates, ventral view **33** supra-anal plate and paraprocts, ventral view **34** subgenital plate, dorsal view **35** hook-like phallomere **36** median phallomere **37** right phallomere. Scale bars = 1.0 mm (Fig. 28), 2.0 mm (Figs 29–30), 0.5 mm (Figs 31–37).

Length, male, pronotum: length × width: 3.0 × 4.0mm, tegmen: 13.0mm, overall length (including tegmen): 15.5mm. Body yellowish brown (Fig. 5). Vertex brown. Ocellar spot pale yellow. Antenna with base yellowish brown and apex dark brown. Maxillary palpomus with fourth and fifth segments dark brown, others yellowish brown (Fig. 6). Pronotum with reddish brown maculae at disk, lateral borders and anterior margin yellowish brown (Figs 5, 28).

Vertex with interocular space slightly less than ocellus width and distinctly narrower than distance between antennal sockets. Third and fifth maxillary palpomus about same length, both distinctly longer than the fourth (Fig. 6). Pronotum with hind margin slightly produced in the middle. Tegmen with apical posterior branch behind the radius vein, which bifurcated at apical part; median vein with two branches, the longer one further bifurcated (Fig. 29). Hind wing with radius vein branched not over the middle, median vein simple, cubitus veins slightly curved with 2 complete and 2 incomplete branches, and triangular apical area reduced and small (Fig. 30). Anteroventral margin of front femur type B_3_, pulvilli present on 4 proximal tarsomeres, tarsal claws symmetrical and unspecialized, and arolia present. First abdominal tergum (T_1_) specialized with a tuft of agglutinated hair directed caudad; seventh abdominal tergum (T_7_) specialized with a pair of large depressions where some setae are situated (Fig. 31); and ninth abdominal tergum (T_9_) with lateral plates similar, not produced and unspecialized, but the right one slightly narrower than the left (Fig. 32).

**Male genitalia.** Male supra-anal plate (Fig. 33) symmetrical with some setae scattered in ventral view, hind margin nearly triangular and slightly concave in the middle. Right and left paraproct dissimilar and unspecialized (Fig. 33). Subgenital plate (Fig. 34) asymmetrical, apex of lateral borders thickened, middle of hind margin slightly produced where two preapical styli are lying; the styli dissimilar, the left one larger, nearly spherical and apex with 3–4 teeth, the right smaller, nearly cylindrical and apex with 2 processes (Fig. 34). Hook of left phallomere with sclerotized portion very small, on left side, hook portion slender and with V-shaped and subapical incision (Fig. 35); median phallomere slightly curved at apical half and apex spine-like and acute (Fig. 36); right phallomere irregular sclerite (Fig. 37).

#### Materials examined.

*Holotype*, male, China: Guangxi Prov., Jinxiu, Mt. Shengtangshan, 18 October 1999, coll. Mingai Gao. *Paratypes*, one male, China: Guangxi Prov., Jinxiu, Luoxiang, 18 October 1999, coll. Xingke Yang; one male, China: Guangxi Prov., Jinxiu, Mt. Shengtangshan, 18 October 1999, coll. Mingai Gao; one female, China: Guangxi Prov., Jinxiu, Mt. Shengtangshan, 18 October 1999, coll. Xuezhong Zhang.

#### Remarks.

The species is similar to *Episymploce mamillatus* (Feng & Woo), but can be distinguished as follows: 1) hind margin of anal plate with indistinct incision (hind margin of anal plate with distinct V-shaped concavity in *Episymploce mamillatus*); 2) right and left paraprocts unspecialized (paraprocts specialized in *Episymploce mamillatus*); 3) subgenital plate with two nearly spherical styli (styli spine-like in *Episymploce mamillatus*). And the species differs from all other *Symploce* spp. by the special styli.

#### Etymology.

The specific epithet “sphaericus” is derived from Latin, which refers to the left style being nearly spherical (dissimilar from the right style).

### 4. Symploce paramarginata sp. n.

http://zoobank.org/66C616E4-5D66-428F-9F29-6C94E374B0EF

http://species-id.net/wiki/Symploce_paramarginata

Figs 7–8 , 38–46

#### Description.

**Figures 38–46.**
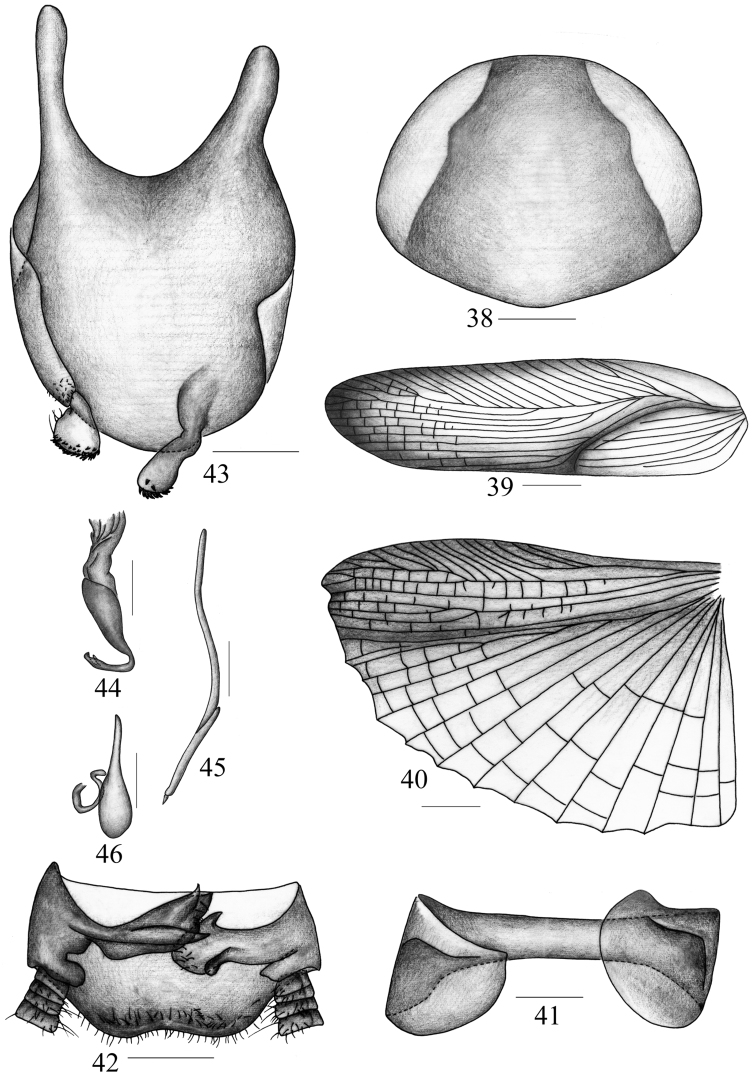
*Symploce paramarginata* sp. n. **38** pronotum **39** tegmen **40** hind wing **41** abdominal tergum 9 and lateral plates, ventral view **42** supra-anal plate and paraprocts, ventral view **43** subgenital plate, dorsal view **44** hook-like phallomere **45** median phallomere **46** right phallomere. Scale bars = 1.0 mm (Fig. 38), 2.0 mm (Figs 39–40), 0.5 mm (Figs 41–46).

Length, male, pronotum: length × width: 3.5 × 4.0mm; tegmen 13.0mm; overall length including tegmen 14.5–16.5 mm. Body dark brown (Fig. 7), vertex and ocellar spot reddish brown and face black. Antenna and maxillary palpomus dark brown (Fig. 8). Pronotum with disc black and lateral borders orange (Figs 7, 38). Tegmina brown, legs reddish brown and abdomen reddish or blackish brown (Fig. 7).

Vertex with interocular space distinctly wider than distance between antennal sockets, and ocellus width slightly less than distance between antennal sockets. Third and fourth maxillary palpomus about same length, both distinctly longer than the fifth. Tegmen with apical posterior branch behind radius vein, and the branch bifurcated, one of the second division further branched (Fig. 39). Hind wing with radius vein branched over the middle and the branches bifurcated at apex; both median and cubitus veins slightly curved, median vein simple, but cubitus veins with 2-3 complete and 1-4 incomplete branches, and triangular apical area reduced and small (Fig. 40). Anteroventral margin of front femur type B_3_, pulvilli present on 4 proximal tarsomeres, tarsal claws symmetrical and unspecialized, and arolia present. First abdominal tergum (T_1_) specialized with a tuft of hair; seventh abdominal tergum (T_7_) unmodified; and ninth abdominal tergum (T_9_) with lateral plates similar, not produced and unspecialized (Fig. 41).

**Male genitalia.** Supra-anal plate (Fig. 42) symmetrical and trapeziform, hind margin slightly concave. Right and left paraproct (Fig. 42) dissimilar, right paraproct with spine-like process on the base. Subgenital plate asymmetrical, left side thickened and upturned, right side slightly upturned; two styli similar and padlike, both apices with minute spines (Fig. 43). Hook of left phallomere large and robust at apex and slender at hook portion, on left side, with V-shaped and subapical incision (Fig. 44); median phallomere distinctly curved at middle and lanciform, and apex spine-like and acute (Fig. 45). right phallomere (Fig. 46) same to that of *Symploce torchaceus*.

Female is similar to male; supra-anal plate symmetrical and trapeziform; subgenital plate simple and hind margin round.

#### Materials examined.

*Holotype*, male, China: Guizhou Prov., Maolan, 24 May 1998, coll. Zizhong Li. *Paratypes*, two males, China: Guangxi Prov., Huaping, 6 November 1963, coll. Jikun Yang; one male, China: Hainan Prov., 25 October 1997, coll. Maofa Yang; one male, China: Guangxi Prov., Jinxiu, 13 May 1999, coll. Xingke Yang; two females, China: Guangxi Prov., Napo, 19 October 2000, coll. Wenzhu Li & Jun Chen; two females, China: Guangxi Prov., Cheng Fang, 25 November 1999, coll. Yanzhou Zhang.

#### Remarks.

The new species superficially resembles *Episymploce marginata* Bey-Bienko, but can be distinguished from the latter by: 1) seventh abdominal tergum unmodified, the latter with two broad sockets covered with hair, 2) subgenital plate with two appendages which is not bifurcated, the latter with apex of appendage bifurcated.

Based on type of vein, the unmodified seventh tergum and symmetrical supra-anal plate, this species should be placed in *Symploce*, and the species is different from other species in this genus for it’s dark brown colors and special macula on pronotum.

#### Etymology.

The Latin word “paramarginata” refers to the superficial resemblance of this species to *Episymploce marginata* Bey-Bienko.

### 5. Symploce evidens sp. n.

http://zoobank.org/CFD981C4-9590-4294-BC9A-C73EDABF8DDF

http://species-id.net/wiki/Symploce_evidens

Figs 9–10 , 47 –58

#### Description.

**Figures 47–55.**
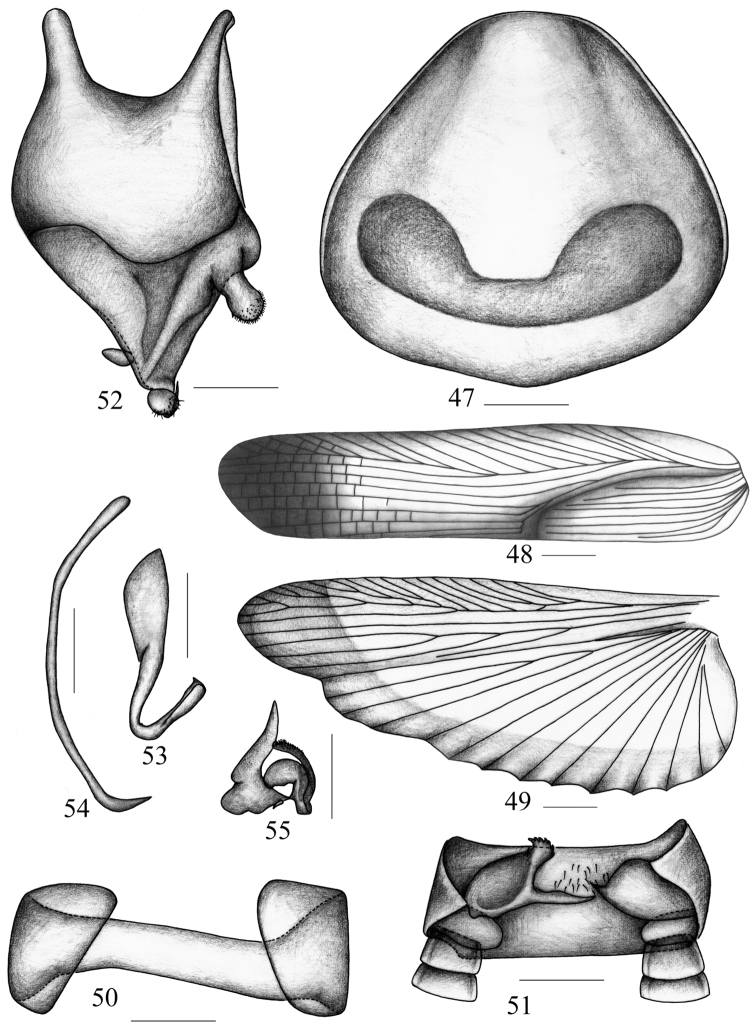
*Symploce evidens* sp. n. **47** pronotum **48** tegmen **49** hind wing **50** abdominal tergum 9 and lateral plates, ventral view **51** supra-anal plate and paraprocts, ventral view **52** subgenital plate, dorsal view **53** hook-like phallomere **54** median phallomere **55** right phallomere. Scale bars = 1.0 mm (Fig. 47), 2.0 mm (Figs 48–49), 0.5 mm (Figs 50–55).

**Figures 56–58.**
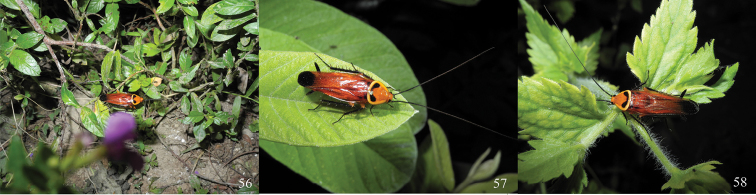
*Symploce evidens* sp. n. in Mountain Qixianling, Baoting County, Hainan Province, 2 May 2013 (photographs by Keliang Wu).

Length, male, pronotum: length × width: 5.5 × 6.0mm, tegmen 18.5–19.0mm; overall length (including tegmen) 24.0mm. Body yellowish brown (Figs 9, 56–58). Antenna pitch-black except apex of flagellum reddish brown. Labrum, maxillary palpus and labial palpus pitch-black. Pronotum yellowish brown with dark brown maculae at disc (Figs 9, 47). Tegmen pale reddish brown and apex blackish brown (Figs 9, 56–58); wing pale brown with inner and apical margin blackish brown (Fig. 49). Legs yellowish brown, tibiae and tarsi blackish brown (Fig. 10).

Vertex with interocular space wider than distance between antennal sockets, and ocellus width about same as distance between antennal sockets. Third and fourth maxillary palpomus about same length, both distinctly longer than the fifth. Pronotum nearly trapezoid with shallow U-shaped macula near base, and hind margin distinctly curved (Figs 9, 47, 56–58). Tegmen with apical posterior branch of radius vein unbranched; median vein also unbifurcated (Fig. 48). Hind wing with radius vein branched beyond the middle, median vein simple, cubitus with 2 complete and 3 incomplete branches, and triangular apical area reduced and small (Fig. 49). Middle of the first abdominal tergite elevated, with a tuft of hair directed forward, T7 and T9 unmodified (Fig. 50).

**Male genitalia.** Supra-anal plate (Fig. 51) symmetrical, hind margin truncate and turning ventrad. Paraprocts dissimilar, right one bearing a long spine-like process and a hand-like process on distad; left one with a spine-like process distad (Fig. 51). Subgenital plate (Fig. 52) asymmetrical, hind margin produced in the middle, right stylus arising at apex and left stylus spine-like directed downwards near base of right one, right side with an irregular sclerite with apex is serrated. Hook-like phallomere on the left (Fig. 53), median phallomere with tapering apex (Fig. 54); right phallomere with lots of irregular sclerites and one of them with slim setae (Fig. 55).

Female is similar to male. Supra-anal plate symmetrical, triangular; subgenital plate broad, hind margin slightly arced, near lateral sides concaved.

#### Materials examined.

*Holotype*, male, China: Hainan Prov., Mt. Jianfengling, Tianchi, 8-10 October 1964, coll. Hui Ren. *Paratypes*, one male, China: Hainan Prov., Mt. Jianfengling, 22 December 1982, coll. Zhiqing Chen; one female, China: Hainan Prov., Mt. Diaoluoshan, 12 October 1965, coll. Sikong Liu; one female, China: Fujian Prov., Huangken, 17 November 1980, coll. Bangkan Huang; one female, China: Hainan Prov., Mt. Jianfengling, Tianchi, 28 December 1983, coll. Tianyuan Luo; one male and one female, China: Hainan Prov., Mt. Jianfengling, 27 April 2013, coll. Shunhua Gui; one male, China: Hainan Prov., Mt. Qixianling, 2 May 2013, coll. Yan Shi; two females, China: Hainan Prov., Mt. Qixianling, 2 May 2013, coll. Yan Shi..

#### Remarks.

This species resembles *Symploce striata*, but can be differed by the following characters: 1) pronotum with shallow U-shaped macula, without macula in *Symploce striata*; 2) apex of tegmen with blackish brown macula, without macula in *Symploce striata*; 3) terminal half of subgenital plate distinctly triangular, trapezoidal in *Symploce striata*.

#### Etymology.

The specific name is derived from the Latin adjective “evidens”, referring to the pronotum with an evident shallow U-shaped macula.

## Discussion

The small order – Blattodea has been investigated and researched for more than two hundred years (Wang and Che 2010). Why are there still more unknown species? The main reason might relate to diversity of habitats, and the methods we used in investigation. In the past time we usually collected cockroaches by searching the habitat for Blattodea by day or at night, especially under dry branches and fallen leaves, or rotting logs, and before obtaining specimens periods of observation may be preceded. This collection tends to be more specific and less ineffective. Passive collecting, such as light traps, also tends to be ineffective. For instance, the most cockroach specimens were obtained by light traps, but species concerned was few; occasionally some were sampled by sweep net, similarly, a narrow variety of cockroaches could be collected.

Traditional sweep at ground litter by day well known by most of cockroach researchers, few collectors can search for cockroaches at night with the aid of highlight torches or cap-lamp, and fewer has a wide knowledge that cockroaches also live on the trees. To everyone’s surprise, Blattodea represented most of the biomass in the canopy (Basset 2001). We have obtained a great number of cockroach specimens by night searching and canopy fogging in recent years. One new species of this paper, *Symploce evidens* sp. n.,only several specimens were acquired by traditional collection after years of effort; but by night searching, we have got a large number of specimens of this species (some soaked in alcohol are excluded) and ecological photos (Figs 56–58) have also been taken successfully. They are mostly secretive and typically ground-dwelling insects that hide by day in cracks and crevices, or under stones (Wang ZQ, pers. obs.). Although having tegmina and well-developed wings, *Symploce evidens* sp. n., usually like to crawl on the leaves and fly slowly at night; even frightened by light or sound, they will not flee in panic like *Periplaneta americana* Linnaeus (Wu KL, pers. obs.).

Adult cockroaches usually have two sets of wings.The tegmina are somewhat sclerotized; while the hindwings are membranous, and generally wider than tegmina. It is generally believed that cross veins play an important role in supporting and reinforcement of the hindwings. Cross veins are normally present throughout most of the wings of cockroaches, and it is only in certain of the more specialized forms such as the Ectobiinae, Anaplectinae and *Oulopteryx* in Corydiidae that they are reduced to a number that can be used in classification (Rehn 1951).

## Supplementary Material

XML Treatment for Symploce

XML Treatment for Symploce torchaceus

XML Treatment for Symploce bispot

XML Treatment for Symploce sphaerica

XML Treatment for Symploce paramarginata

XML Treatment for Symploce evidens
